# Relationship of selected conditioning parameters and sport performance indicators in karate

**DOI:** 10.3389/fspor.2024.1433117

**Published:** 2024-07-12

**Authors:** Kristina Nema, Pavel Ruzbarsky, Łukasz Rydzik, Tomas Peric

**Affiliations:** ^1^ Department of Educology of Sports, Faculty of Sport, University of Presov, Presov, Slovakia; ^2^ Department of Sports Kinantropology, Faculty of Sport, University of Presov, Presov, Slovakia; ^3^ Institute of Sports Sciences, University of Physical Education in Krakow, Krakow, Poland; ^4^ Department of Sports Educology and Humanities, Faculty of Sport, University of Presov, Presov, Slovakia

**Keywords:** combat sports, aerobic performance, anaerobic performance, technical indicators, tactical indicators

## Abstract

**Introduction:**

The variables of aerobic performance and aerobic capacity are of significant importance in maintaining intensity during a fight and also contribute to faster recovery between rounds in sports fighting in karate. Anaerobic performance is crucial for the execution of the techniques during high-intensity offensive or defensive actions that determine the outcome of a fight. The objective of this study was to assess the relationship between selected performance indicators of aerobic and anaerobic capacity to sports performance in karate.

**Methods:**

The study included six male karate athletes (age 28 ± 3 years, body mass 85.10 kg; height 185.5 cm), medalists from European and World championship, events in senior categories. The selection criteria included training experience and sports level. The Karate specific aerobic test (KSAT) was use in conjunction with heart rate monitoring and changes in blood lactate levels to diagnose special aerobic endurance parameters. To determine the level of anaerobic performance the Wingate test were choosed. Technical and tactical indicators (efficiency, effectiveness and activeness of the attack) were used to assess the sports skill level during competition.

**Results:**

Based on the Spearman correlation coefficient results demonstrated statistically significant differences (r_s_ = −0.81) with large effect size index between efficiency of the attack and average heart rate achieved in the test KSAT. Additionally statistically significant differences (r_s _= 0.81) with large effect size were demonstrated between the fatigue index and efficiency of the attack Furthermore, the selected indicators of special aerobic endurance parameters and anaerobic performance demonstrated a high degree of predictive validity in relation to the efficiency (r_p_ = 0.960) and activity (r_p_ = 0.927) of attacks.

**Conclusion:**

The high level of predictive validity confirmed the importance of a high level of anaerobic conditions for performance in karate. The low values of the average heart rate in relation to the efficiency of the attack confirm the high performance level of karate athletes in relation to special aerobic performance parameters. It was found that the effectiveness of the attack had no relation to the monitored parameters.

## 1 Introduction

In the contemporary era, karate is widely regarded as a combat sport, wherein the traditional tenets of the martial art are blended with the tenets of a modern fighting sport. Research into this sport has demonstrated that fighting in karate is typified by a high-intensity acyclic activity comprising bief, intense actions interspersed with periods of low-intensity rest (1). The effort expanded a karate match is not uniform; rather, it can vary in flow, rhythm and pace depending on whether the referee has interrupted it or not. Due to the intermittent nature of kumite, the total energy cost in a fight is approximately paid by the aerobic component (70%), with the utilisation of alactic energy stores (20%) and lactic acid production (10%) (2). Similar findings were also recorded in research by Beneke et al. (3), which demonstrated that from a physiological perspective, aerobic metabolism is the primary source of energy during the sport of kumite. However anaerobic supplementation and high-energy phosphates also contribute to this process. Nevertheless, it is possible that the aforementioned reactions may differ between official and simulated matches. A number of studies have indicated that the overall metabolic profile is predominantly aerobic, although anaerobic processes exert a critical influence (2–4). Consequently, it has been proposed that in order to achieve a high level of competitive performance, karate athletes must develop both anaerobic and aerobic fitness (2).

It is important to consider both aerobic capacity and aerobic power when evaluating an athlete's fitness. Aerobic power allows an athlete to maintain a high intensity for longer periods during a match, while aerobic capacity contributes to faster recovery between consecutive matches. The traditional approach to evaluating the aerobic fitness of karate athletes has involved the use of laboratory treadmill and cycle-ergometer tests to determine maximal oxygen uptake. Although laboratory tests are accurate, their lack of sport specificity is a significant criticism of these assessment methods (5). To assess specific aerobic performance in karate, the Karate-specific aerobic test (KSAT) was developed (6), which, according to Chaabene et al. (7), can effectively distinguish between different levels of karate practitioners, for example, national vs. regional level. The study by Tabben et al. (5) demonstrated that the KSAT was a reliable assessment tool, with oxygen consumption values recorded during the KSAT exhibiting a strong correlation with those obtained from the cycle ergometer test (*r* = 0.81; R2 = 65%). Furthermore, the time to exhaustion during the KSAT displayed a very large correlation with VO2max from laboratory tests (R2 = 65%).

Anaerobic performance is crucial for the execution of techniques during high-intensity attacking or defensive actions that are responsible for scores during a match. Conversely, anaerobic capacity is considered less important for karatekas because the duration of high-intensity actions is relatively brief. Given the absence of karate-specific anaerobic tests, the majority of research has employed the Wingate test to assess anaerobic performance. This approach has been adopted by numerous studies (2, 8–11). According to Zemková (8), maximum power at the beginning of the load refers to the ability to mobilise energy in a very short period of time, which expresses the level of maximum anaerobic power. This capacity to generate maximum power in brief time intervals is employed primarily in offensive and defensive actions. The average performance, defined as the total work done in 30 s, is indicative of the capacity to resist fatigue during an intense short-term load, such as a karate match. It is a measure of maximum anaerobic capacity.

To assess anaerobic endurance, the fatigue index is employed as an additional parameter, representing the decline in performance expressed as a percentage from the initial to the final phase of the load (5, 8, 12, 13). An alternative approach is to quantify the load intensity through the use of appropriate indicators of the body's response to loads. The most commonly employed indicator is the level of blood lactate in the blood, which is highly responsive to the load, during which the organism is compelled to meet a portion of its energy needs anaerobically.

The objective of this study is to examine the relationship between selected aerobic and anaerobic parameters and sports performance indicators in karate. The study's objective was to formulate a research question. This question was: What is the relationship between the levels of aerobic and anaerobic parameters (assessed through KSAT and WAnT) and the levels of specific performance indicators in karate?

### 1.1 Hypothesis

There is a significant relationship between selected aerobic and anaerobic parameters and sports performance indicators in karate. Specifically, lower average heart rate values and higher fatigue index values are associated with higher attack efficiency in karate.

## 2 Material and methods

### 2.1 Participants

The study included 6 male karate athletes from Poland, with an average age of 28 ± 3 years. According to the results of the previous competition season, the karate athletes were included in the senior categories at the international level. They had fought an average of 15 fights per year in national and international tournaments and their training experience depending on their age was an average of 9 ± 1 years. They were also medalists in European and World Championship. The weekly training load of the karate athletes was 11 ± 1 h of training load. Inclusion criteria for the study were: sport discipline kumite, at least 5 years training experience and the sport level as assessed by authors' observation and coach's opinion, active competitor, uninterrupted training process for at least 6 months before inclusion in the study, no musculoskeletal injuries, gender, success in international competitions. Exclusion criteria were: injuries, gender, a training experience of less than 5 years, no competition results. The anthropometric characteristics of the karate athletes presented were: body mass 85 ± 10 kg, height 185.5 ± 6.50, body mass index 24.95 ± 0.95 kg.m^−2^, body fat percentage 17.2 ± 3.35% and free fat mass index 70.00 ± 3.30 kg. Body composition was assessed using a Tanita BC-601 whole-body bioimpedance analyzer.

### 2.2 Diagnostics of aerobic and anaerobic parameters

To diagnose the level of aerobic parameters, was used a specific test by Nunan (6)- the Karate specific aerobic test (KSAT). The test consists of a combination of the most frequently used punches and kicks in karate (direct punch with the front hand—kizami tsuki, arc kick with the back leg—mawasi geri, direct punch with the back hand gyaku-tsuki, arc kick with the back leg—kiza mawasi geri), which the subject has to perform repeatedly within 7 s. This test is based on the principle of the beep test, where the time to perform the combination is not changed, but the length of the pause is changed, i.e., it is shortened. Time to exhaustion was used as the performance parameter in the KSAT test. The validity and reliability of the test have been verified in several studies (5, 7, 14).

Maximum and average heart rate recorded using Polar Team Pro (Polar Electro Inc., New York, USA) were choosen as an auxiliary indicators to monitor the intensity of the load during the KSAT.

The internal response of the body karate athlete's body to the completed exercise was assessed by monitoring changes in blood lactate (La) levels. Biochemical analysis of blood samples was performed using a Biosen C-line Clinic device (EKF Diagnostic GmbH., Cardiff, UK). Absolute changes and percentage decreases at specified time intervals were evaluated. Lactate clearance rate was determined as the difference between the subjects' blood lactate concentration at 5 and 15 min of recovery.

The Wingate test using the Cyclus 2 bicycle ergometer (RBM elektornik- automation Gmbh., Leipzig, Germany) was chosen for the diagnosis of anaerobic parameters. It is a test that is most commonly used to assess the anaerobic capabilities of karate athletes (2, 8, 9). The performance parameters considered in the Wingate test were peak power, average power, anaerobic power, anaerobic capacity, average power and the fatigue index, which expresses the decrease in power during the test (IU). The mechanical resistance was set at 7.5% of the subject's body weight, based on the literature (15, 16). The test was performed according to the protocol described by the authors InBar O., Bar Or O., Skinner J. S (17).

Diagnosis of the selected tests was carried out over two days during one week, with a two-day break in between. On the first day, the karate athletes completed the Wingate test and body composition measurement. On the second day, the karate athletes completed the Karate specific aerobic test. Both tests were performed at the same time of the day, under the same conditions and constant room temperature (21°C). Prior to the tests, they were warned not to use any prohibited substances aids or medications, not to eat heavy meals for 12 h before the test, and not to perform any physically demanding activities. They were also warned to take a day off before the test and to get enough sleep.

### 2.3 Measuring the indicators of technical and tactical training

In order to determine the level of athletic skill during the competition, the fights were analysed and appropriate calculations were made. Three fights of each karate athlete were analysed. The fights were supervised by two experts with karate coaching qualifications. The researchers recorded the data on special spreadsheets. The results were summarised, and the average of two records was taken. The analysis of each round was based on a digital recording of the fight. The indicators of technical and tactical training were then determined using the following formulas (18–21).

Efficiency of the attack (S_a_)

$$S_{a}=\frac{n}{N}$$

N- Number of bouts.
*n*- Number of attacks awarded 1 pt.

Effectiveness of the attack (E_a_)

$$E_{a}=\frac{\mathrm{number}\;\mathrm{of}\;\mathrm{effective}\;\mathrm{attacks}}{\mathrm{number}\;\mathrm{of}\;\mathrm{all}\;\mathrm{attacks}}\times 100$$

* An effective attack is a technical action awarded a point.
* Number of all attacks is the number of all offensive actions.

Activeness of the attack (A_a_)

$$A_{a}=\frac{\mathrm{number}\;\mathrm{of}\;\mathrm{all}\;\mathrm{registred}\;\mathrm{offensive}\;\mathrm{actions}\;\mathrm{of}\;a\;\mathrm{karate}\;\mathrm{athletes}}{\mathrm{number}\;\mathrm{of}\;\mathrm{bouts}\;\mathrm{fought}\;\mathrm{by}\;a\;\mathrm{karate}\;\mathrm{athletes}}$$

### 2.4 Bioethical committee

The study was conducted according to the tenets of the Declaration of Helsinki (22). Written informed consent was obtained as a prerequisite for participation in the project. All subjects gave informed consent for invasive capillary blood sampling. The study was approved by the Bioethics Committee at the Regional Medical Chamber (No.287/KBL/OIL/2020).

### 2.5 Statistical analysis

Statistical analysis of the data was performed using Statistica 13.5 (Tibco Software Inc., California, USA). Statistical analysis was performed using non-parametric tests due to the small number of research samples. The normality of the distribution was checked using Shapiro-Wilk test, which proved the normality of the studied variables. Spearman's correlation coefficient (r_s_) was used to determine the relationship between selected aerobic and anaerobic parameters and sports performance, with the level of statistical significance set at *p* < 0.05. The evaluation of ES index r was interpreted as 0.10 ≤ *r* 0.29—small effect, *r* = 0.30 ≤ *r* 0.49—medium effect, *r* ≥ 0.50—large effect based on Cohen (23).

The determination of the validity of the prediction of the efficiency and activeness of the attacks was based on exploration using the correlation coefficient. The calculation of the predictive validity was based on a regression analysis, where three indicators (average heart rate, anaerobic power and fatigue index) were included as independent variables and the efficiency of the attack and the activeness of the attack were used as dependent variables.

## 3 Results

The results of indicators of specific aerobic endurance parameters together with indicators of anaerobic power parameters are presented in Table 1. The results of the efficiency, effectiveness and activeness of the attack are also shown in Table 1.

**Table 1:** Monitored parameters.

Variables		Karate athletes (*n* = 6)	
		Med	QD
Indicators of special aerobic endurance parameters	KSAT [s]	904.00	28.50
	Maximal HR [bpm]	196.00	8.00
	Average HR [bpm]	177.00	6.50
	LCR [mmol/L]	3.94	0.23
	Lactate after 3 min. [mmol/L]	12.07	2.14
Indicators of anaerobic power parameters	Peak power [W]	951.15	49.60
	Average power [W]	733.20	35.60
	Anaerobic power[W.kg^−1^]	10.60	0.15
	Anaerobic capacity[W.kg^−1^]	8.60	0.15
	Fatigue index [%]	44.88	9.88
	Average force [N]	369.50	30.00
Indicators of technical and tactical training	Efficiency of the attack	8.00	1.25
	Effectiveness of the attack	17.74	2.08
	Activeness of the attack	47.75	11.25

KSAT, karate specific aerobic test; Maximal HR, maximal heart rate; Average HR, average heart rate; LCR, Lactace clearance rate; QD, quartile deviation; Me- median.

Based on the results of the Spearman correlation coefficient, statistically significant differences were found between efficiency of the attack and fatigue index (*p* = 0.05). The effect size was evaluated as large (−0.81). It is also possible to observe the occurrence of dependence between efficiency of the attack and average heart rate obtained in the KSAT test. This correlation was statistically significant at *p* < 0.05. The effect size was considered to be large (0.81). Based on the determination of the critical value, the other correlations were not statistically insignificant (Table 2).

**Table 2:** Relationship between indicators of technical and tactical training and the results of fitness test.

Karate athletes		Indicators of technical and tactical training					
		Efficiency of the attack		Effectiveness of the attack		Activeness of the attack	
		*p*	*r*	*p*	*r*	*p*	*r*
Indicators of special aerobic endurance parameters	KSAT [s]	0.96	0.03	0.21	0.60	0.47	−0.37
	Maximum HR [bmp]	0.26	0.54	0.35	0.46	0.91	0.06
	Average HR [bmp]	0.05*	**−0**.**81**^b^	0.79	0.14	0.16	−0.66
	LCR [mmol/L]	0.29	0.52	0.96	0.03	0.54	0.31
Indicators of anaerobic power parameters	Peak power [W]	0.26	0.55	0.87	0.09	0.79	0.14
	Average power [W]	0.70	−0.20	0.70	0.20	0.54	−0.31
	Anaerobic power [W.kg^−1^]	0.20	0.60	0.46	−0.38	0.06	0.78
	Anaerobic capacity [W.kg^−1^]	0.39	−0.43	0.65	0.23	0.57	−0.29
	Fatigue index [%]	**0**.05*	**0**.**81**^b^	0.47	−0.31	0.11	0.71
	Average force [N]	0.83	−0.12	0.87	0.09	0.54	−0.31

KSAT, karate specific aerobic test; Maximal HR, maximal heart rate; Average HR, average heart rate; LCR, lactace clearance rate; r = Spearman's correlation coefficient and effect size (0.1—small; 0.3—medium; 0.5^b^—large); *p* = statistical significance *—*p* < 0.05.

Bold indicates values that are statistically significant.

To estimate the importance of each variable, three indicators with the highest correlation value (average heart rate, anaerobic power and fatigue index) were selected, which were entered into the regression equation (Table 3). The results of the regression statistic was for the efficiency of the attack: multiplied *R* = 0.96, reliability value *R* = 0.92. And for the activeness of the attack was multiplied *R* = 0.93, reliability value *R* = 0.86. The predictive validity of these indicators was calculated for the selected performance parameters.

**Table 3:** Regression statistic.

	Efficiency of the attack	Activeness of the attack
Constant	−4.30	−278.37
Average heart rate	−0.10	0.39
Anaerobic power	2.67	18.04
Fatigue index	0.10	4.23

The determination of the predictive validity of the efficiency and activeness of attacks was calculated using a correlation based on the predicted values from the regression equation and the results obtained in the given variable. The predictive validity was 0.96 for the efficiency of the attack and 0.93 for the activeness of the attack. It turned out that the effectiveness of the attack was no related to the monitored parameters.

## 4 Discussion

The primary findings of the research indicate statistically significant differences between the efficiency of the attack and the fatigue index, as well as between the efficiency of the attack and the average heart rate achieved in the KSAT test. The high level of predictive validity confirmed the importance of maintaining a high level of anaerobic conditions for optimal performance in karate.

In the sport fight- kumite, as in other combat sports, the primary scoring criterion is the intensive use of punches and kicks (24). Therefore the technical and tactical performances during kumite depend on physical qualities (25), such as the maximum speed, explosive power and special endurance (9, 26–28). The results of the special aerobic performance parameters, expressed as time to exhaustion in the KSAT test, reached a median of 904 s. This can be interpreted based on the similarity of the research samples as a high level of special endurance, which is comparable to the results of other authors (26, 29). In the study by Silva et al. (29), seven karate athletes with a technical level of 1 Dan and above achieved a mean time of 438.43 ± 178.04 s in the test. by Chaabene et al. (26) monitored the performance of 43 karate athletes with a technical level of 1 Dan and above, categorised according to their performance levels. The karate athletes in the study were of a similar age to the general population, with an average age of 24.1 ± 4.7 years and with a sports age of 9 ± 5.2 years. They trained four times a week for 2 h and achieved a mean time of 841 ± 134 s. A second group of 19 karate athletes, aged 25.6 ± 3.3 years and with a sports age of 7 ± 4.4 years achieved 871 ± 150 s in the test and 881 ± 158 s. They performed the test twice, with a weekly interval.

The average and maximum heart rate values observed in our study were comparable to those observed during simulated and official fights at the national championship (26), where high-performance karate athletes achieved maximum heart rate values of 193 ± 8 bpm and 192 ± 9 bpm for official and simulated fights. The average heart rate was 177 ± 13.43 bpm (91.70% SF_max_) for simulated fights and 175 ± 11 bmp (91.14% SF_max_) for official fights. These findings indicate that the KSAT is an appropriate instrument for assessing the level of special endurance in karate athletes. Similarly, the results of the maximum heart rate with a value of SF_max_ = 196 ± 11 bmp were also found in the research by Tabben et al. (5), when 17 karate athletes at an international level performed the KSAT test. Hausen et al. (30), in their research to assess the cardiorespiratory fitness of national level karate practitioners, used a different test, namely the Graded Karate Test, but it turned out that the performance of karate practitioners in this test was similar to the performance of karate practitioners in this research. Additionally, it was demonstrated that there were statistically significant differences between the efficiency of the attack and the average heart rate achieved in the KSAT test. The observed differences were statistically significant at the 0.05 level. The low values of the average heart rate in relation to the efficiency of the attack, indicate that karate athletes exhibit a high level of aerobic endurance. This was also corroborated by the high degree of predictive validity, efficiency, and activeness of the attack. It is possible that certain tendencies may indicate a higher correlation coefficient between maximum heart rate and technical tactical indicators. However, this was not statistically significant due to the limited size of the research sample.

A comparison of our findings with those of other combat sports reveals that the maximum heart rate values observed inkickboxing (31, 32) and Thai boxing (33, 34) are higher. As undicated by Slimani et al. (35) this may be attributed to the higher technical-tactical and energy demands inherent to karate compared to the aforementioned combat sports.

Following the completion of the KSAT test, the maximum blood lactate concentrations were recorded in the third minute following the conclusion of the test. The findings were consistent with those of Janssen (36) and Shepard (37), who state that after high-intensity exercise, blood lactate levels reach their highest values between 3 and 5 min. The observed correlation between the median of the lactate clearance rate recorded after the KSAT test and technical and tactical indicators suggests that karate who demonstrate superior recovery capabilities may be more adept at executing scored and non-scored attacks during competition. In comparison to the findings of (9), which monitored the concentration of lactate in the blood after 3 min from the end of the KSAT test with karate athletes of the international level, our karate athletes demonstrated a significantly higher concentration of lactate in the blood. In the study by Chaabene (9), the lactate concentration was found to be approximately 6.23 ± 1.03 mmol/L. Similar values as Chaabene (9) were also recorded in other research studies. Beneke et al. (3) reported that the blood lactate concentration of karate athletes following a competitive karate fight was 5.9 ± 1.6 mmol/L. Significantly lower results were observed during a simulated karate fight, with lactate concentrations of 3.4 ± 1 mmol/L (4). The changes in blood lactate levels that occur after exercise are a highly dynamic variable. It is important to consider the possibility of intra-individual variability within the sampling methodology when determining the optimal sampling after loading. From a methodological standpoint, the disparate outcomes observed in our research may be attributed to the site of blood collection (fingertip). In this context, it was demonstrated that samples collected from the earlobe, as previously observed by Beneke et al. (3), Lide et al. (4), Chaababe (9), exhibited lower blood lactate values than fingertip samples (38). Consequently, given that the blood samples in our research were obtained from the fingertips, it is possible that the lactate concentration determined following KSAT during our research may have been overestimated. The GKT yielded higher blood lactate concentration responses (∼14.6 mmol/L^−1^) (30) compared to the Karate-specific test, a difference that may be explained by the nature of the movements performed in the different protocols or by the sample of training profiles. In the previous proposals, the athletes provided an active displacement between the strikes, whereas the KSAT increased the strike frequency.

The results of the predictive validity of efficiency and activeness of the attack, which monitor the number of scored and non-scored techniques in relation to individual rounds, indicate the importance of anaerobic performance in offensive and defensive actions responsible for scoring during the fight. A comparison of the anaerobic performance achieved in the Wingate test with other research (2, 10, 39), relealed that our karate athletes achieved significantly better results than those observed by Alp and Gorur (39), where the karate athletes under observation achieved values of 6.97 ± 1.54 W/kg. Conversely, comparable results were observed in the other studies (40, 41). In studie of Ravier et al. (10) where national-level karate athletes demonstrated a mean power output of 10.9 ± 1.5 W/kg. Concurrently, these authors also observed karate athletes of an international level with a maximum one-time power of 12.5 ± 1.3 W/kg, while they noted significant differences between the two groups. These results, as reported by Chaabane et al. (26) appear to support the hypothesis that kumite performance is more dependent on anaerobic power than on anaerobic capacity itself. According to Chaabane (9), the aforementioned findings have significant practical implications, particularly when differentiating between karate athletes at varying competitive levels.

The indicative tendency between the median of anaerobic capacity in relation to the efficiency and activeness of the attack could be indicative of the nature of the karate for which the karate athletes are trained and which is characterised by a short duration of high-intensity offensive or defensive actions (3, 9). A comparison of the results of the anaerobic capacity in the Wingate test, similar to the anaerobic performance, revealed that the karate athletes in this study exhibited superior results compared to those of karate athletes from other studies who competed in the sport of kumite. Significantly lower values of average power per kilogram of body weight (5.12 ± 0.99 watt/kg) were recorded for karate athletes in the research of Alp and Gorur (39). This result was comparable to that observed by Sanchez-Puccini et al. (42), where 19 karate athletes of international level also recorded lower values of anaerobic capacity (4.8 ± 0.9 watt/kg). Doria et al. (2) recorded similar results of 7.9 ± 0.6 watt/kg in three karate athletes of international level (medalists from the World and European Championships) with an average age of 24 ± 4.6 years. Similar results of 8.75 ± 0.15 watt/kg were found in the study of Nema and Ruzbarsky (40).

A comparison of the values of the maximum and average performance related to the body weight of the karate athletes in our research group with those of fighters in similar combat sports, such as taekwondo (39, 43), Judo (44), MMA (45) or kickboxing (46), reveals karate athletes achieved similar results. The anaerobic power of taekwondo athletes was found to be 7.3 ± 0.68 watt/kg (43), 9.26 ± 2.4 watt/kg (39), that of MMA athletes ranged from 9.8 to 10.4 watt /kg (45) and that of kickboxers was 10.5 watt/kg (46), 11.4 ± watt/kg (47). The anaerobic capacity of taekwondo athletes was found to be 5.12 ± 0.99 watt/kg (39), while that of MMA athletes was between 7.5 and 7.9 watt/kg (45). Finally, the anaerobic capacity of kickboxers was found to be 7.82 ± 0.57 watt/kg (46).

The median value of the fatigue index, which is one of the parameters of anaerobic performance was 44.88%. These values correspond with the range of values for the decrease in performance observed in strength sports athletes at a high level of training, with a decrease of between 44.6% and 53.5% (48). Statistically significant differences were observed between the fatigue index and the efficiency of the attack. This may be attributed to the intermittent nature of kumite, where aerobic metabolism (70%) is the primary source of energy, followed by anaerobic lactate coverage (20%) and anaerobic lactate coverage (10%) (2). To facilitate comparison with other research, Sanchez-Puccini et al. (42) and Doria et al. (2) achieved better results, namely 40.8 ± 4.2 (10) and 34.1 ± 14.1 (40), where the karate athletes were aged between 27 and 35 years old and had participated in the highest international competitions for a minimum of 3 years.

A positive finding was the high level of predictive validity, confirming the importance of a high level of anaerobic prerequisites for performance in karate. Karate can be characterised as a sport with high-intensity intermittent sport. This finding is also based on the findings of other authors (2, 9). This has implications for training planning and the determination of the loading strategy.

### 4.1 Limitations of the study

The main shortcoming of our research is the small research sample of male karate athletes, which limits the generalisability of the findings. However it should be noted that this sample consisted of elite karate athletes, with medals from the European and World Championships. The statistical analysis of technical and tactical indicators evaluated the number of scored and non-scored techniques during the entire fight, we did not verify them during individual rounds of the fight. The technical tactical indicators were calculated on the basis of official fights at competitions, which did not allow us to monitor the heart rate during the fight and the concentration of lactate in the blood after the fight. We also did not compare the values of the technical and tactical indicators with other results, as we were the first to use these indicators for evaluation in karate.

## 5 Conclusions

The primary goal of karate training is to ensure the development of those skills that limit sport performance. Our research assessed the relationship between selected aerobic and anaerobic performance indicators and performance in karate. The results showed statistically significant differences with a large effect size index between the efficiency of the attack and the average heart rate achieved in the KSAT test. The low values of average heart rate in relation to the efficiency of the attack confirm the high level of performance of karate athletes in terms of aerobic endurance. This was also confirmed by the high level of predictive validity of efficiency and activeness of the attack. The results also showed statistically significant differences with large effect size between fatigue index and efficiency of the attack. This can confirm the importance of anaerobic performance during offensive and defensive actions responsible for the scoring during the fight, based on the results of predictive validity efficiency and activeness of the attack, which monitor the number of scored and non-scored techniques in relation to individual rounds. It turned out that the effectiveness of the attack had no relation to the monitored parameters.

### 5.1 Practical implications

In order to be able to design a specific training programme aimed at the development of aerobic and anaerobic metabolism, it is necessary to point out the methods that are usually used to quantify the training load. The present study may indicate a modification of the training process in karate through the development of anaerobic prerequisites, which may affect the improvement of technical-tactical actions in the fight. However, due to the small research group, it would be advisable to carry out more extensive research with a larger number of male and female karate athletes.

## Data Availability

The raw data supporting the conclusions of this article will be made available by the authors, without undue reservation.
